# Integrating Natural Deep Eutectic Solvents into Nanostructured Lipid Carriers: An Industrial Look

**DOI:** 10.3390/ph17070855

**Published:** 2024-06-28

**Authors:** Luísa Schuh, Luane Almeida Salgado, Tathyana Benetis Piau, Ariane Pandolfo Silveira, Caio Leal, Luís Felipe Romera, Marina Arantes Radicchi, Mac-Kedson Medeiros Salviano Santos, Leila Falcao, Cesar Koppe Grisolia, Eliana Fortes Gris, Luis Alexandre Muehlmann, Sônia Nair Báo, Victor Carlos Mello

**Affiliations:** 1 Cooil Cosmetics, Brasília 72622-401, DF, Brazil; luisaschuh.vargas@gmail.com (L.S.); lusalgado08@gmail.com (L.A.S.); pandolfo.ariane@gmail.com (A.P.S.); caio.leal1509@gmail.com (C.L.); lfromera7@gmail.com (L.F.R.); maradicchi.bep@gmail.com (M.A.R.); 2 Laboratory of Nanobiotechnology, Department of Genetics and Morphology, Institute of Biological Sciences, University of Brasília, Brasília 70910-900, DF, Brazil; 3 Laboratory of Microscopy and Microanalysis, Department of Cell Biology, Institute of Biological Sciences, University of Brasília, Brasília 70910-900, DF, Brazil; snbao@unb.br; 4 Laboratory of Genetic Toxicology, Department of Genetics and Morphology, Institute of Biological Sciences, University of Brasília, Brasília 70910-900, DF, Brazil; tathyanabenetis@yahoo.com (T.B.P.); grisolia@unb.br (C.K.G.); 5 Green Nanotechnology Group, University of Brasília, Brasília 72220-900, DF, Brazil; mackedson@hotmail.com; 6 Inaturals SAS, 2 Bis, Impasse Henri Mouret, 84000 Avignon, France; leila.falcao@inaturals.fr; 7 Faculty of Ceilândia, University of Brasília, Brasília 72220-275, DF, Brazil; elianagris@gmail.com (E.F.G.); luismuehlmann22@gmail.com (L.A.M.)

**Keywords:** NLC, NaDES, green nanotechnology

## Abstract

The industries are searching for greener alternatives for their productions due to the rising concern about the environment and creation of waste and by-products without industrial utility for that specific line of products. This investigation describes the development of two stable nanostructured lipid carriers (NLCs): one is the formulation of a standard NLC, and the other one is the same NLC formulation associated with a natural deep eutectic solvent (NaDES). The research presents the formulation paths of the NLCs through completeness, which encompass dynamic light scattering (DLS), zeta potential tests, and pH. Transmission electron microscopy (TEM) and confocal microscopy were performed to clarify the morphology. Cytotoxicity tests with zebrafish were realized, and the results are complementary to the in vitro outcomes reached with fibroblast L132 tests by the MTT technique and the zymography test. Infrared spectroscopy and X-ray diffractometry tests elucidated the link between the physicochemical characteristics of the formulation and its behavior and properties. Different cooling techniques were explored to prove the tailorable properties of the NLCs for any industrial applications. In conclusion, the compiled results show the successful formulation of new nanocarriers based on a sustainable, eco-friendly, and highly tailorable technology, which presents low cytotoxic potential.

## 1. Introduction

Over recent decades, the world has witnessed remarkable advancements that have significantly influenced various economic sectors. These impressive developments, particularly in materials science and nanotechnology, have notably impacted the pharmaceutical and cosmetics industries. The 2023 Start-up Genome Report underscores this trend, highlighting exponential growth in deep tech investments, which include areas such as artificial intelligence, life sciences, and nanotechnology [1,2]. This trend was especially pronounced in the pharmaceutical industry during the SARS-CoV-2 pandemic, where global collaborations underscored the importance of technological advancements. The agility and collaborative nature of these sectors, accelerated by the pandemic’s demands, shed light on the crucial role of cutting-edge technology in addressing global health challenges [2].

Despite this promising landscape, the transition from fundamental scientific research to tangible market innovations remains fraught with significant challenges. A critical aspect of this process is the role of solvents in various industries, making them present in a wide array of everyday products. The growing necessity to develop environmentally friendly technologies has led to the rise of natural deep eutectic solvents (NaDESs) as promising alternatives to circumvent issues associated with traditional solvents. Composed of naturally occurring compounds such as organic acids, sugars, and amino acids, NaDESs offer a less environmentally detrimental approach and are noted for their effectiveness, reduced cost, and ease of large-scale synthesis [3,4].

Parallel to this, the development of nanostructured lipid carriers (NLCs) has marked a significant advancement over solid lipid nanoparticles (SLNs), their precursors. This advancement is characterized by a dual lipid phase structure (solid and liquid), which translates into a greater loading capacity for active compounds and the prevention of potential expulsion of these compounds during encapsulation. Incorporating NaDESs into NLCs, along with native lipids from Brazilian flora such as tucumã butter and jambu oil, promises innovative applications in active delivery systems [5,6].

This work focused on three fundamental stages: developing the first NLC incorporating NaDESs into its composition, assessing biosafety from a toxicity perspective, and presenting a methodology for altering the diameter of the nanocarrier without changing its composition. This approach was aimed at adapting the NLCs for various industrial applications, considering that each application requires an ideal nanoparticle size. Focusing on adapting the technology for diverse applications reflects a significant stride towards industrial flexibility and efficacy, establishing a new paradigm in environmentally friendly, efficient, and broadly applicable nanotechnological solutions.

## 2. Results and Discussion

### 2.1. The Formulation Process

This study reports the development of a new, eco-friendly technology that shows how industrial and laboratorial processes could align with sustainability principles. Natural lipids from the Brazilian flora were used, which are not usually employed in the development of nanostructures, although they have great potential to yield good nanoformulations for cosmetics and foods. Also, all these butters and oils were industrial by-products, providing a new role for materials that were initially considered waste. At the end, the goal was to prove that greater technologies can be made from natural products and by saving water, without harming nature itself and losing efficiency in industries.

In order to achieve this goal, this present work was based in the research of Mello [7] and Mello et al. [8], but with the formulation of a new generation of nanocarriers (NLCs instead of a SLNs) and the addition of NaDESs. NLCs are already known for having properties such as enhanced stability, low toxicity, increased shelf life, improved drug loading capacity, and biocompatibility when compared to SLNs [9]. Also, NaDESs have low cytotoxicity and are biodegradable [4]. At the end, the goal was to create an almost entirely green nanocarrier, and it was successfully accomplished with the formulation of JamaNaDES.

The formulation characterization includes dynamic light scattering tests and TEM microscopy. In vitro tests were performed to study the cytotoxicity of the carrier. It is important to note the difference in pH between Jama and JamaNaDES. The first one has a pH of 4.5, while the second one has a pH of 2.7.

**Figure A1:**
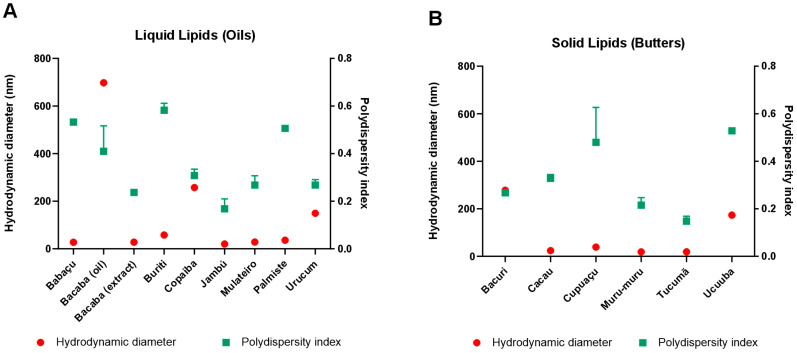
DH and PDI results obtained using the DLS technique in ZetaSizer were evaluated in order to study the behavior of the formulation of different oils and butters. This can be verified in (**A**,**B**), respectively.

The DHs and PDIs of the nanostructures produced with butter or oils alone can be seen in Figure A1A and Figure A1B, respectively. Both were formulated based on a base protocol developed by our research group utilizing Box–Behnken designs, which are experimental projects for the methodology of surface response [7,8]. The similar results of two lipids led to their combination, leading to the mixing of tucumã butter and jambu oil.

**Figure 1:**
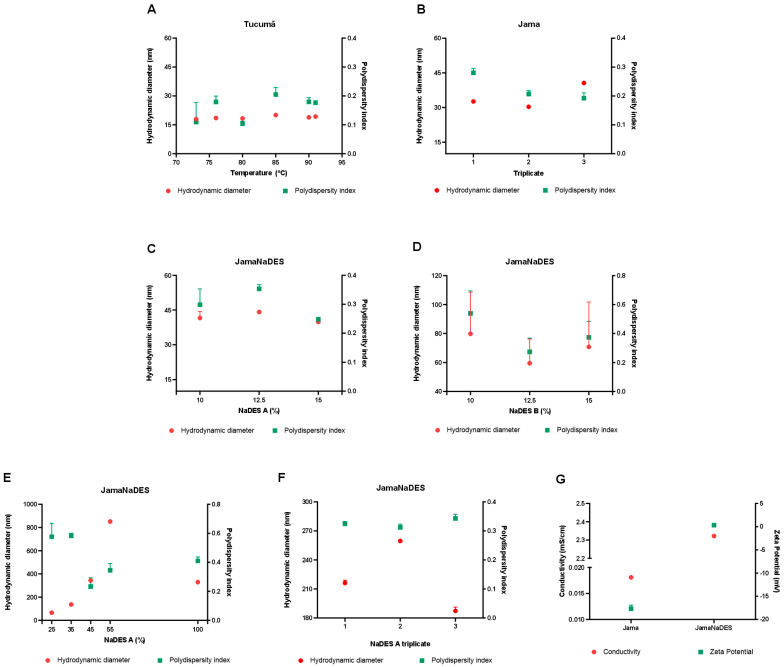
(**A**) shows the temperature test performed with the SLN with just tucumã butter. In (**B**), the addition of the oil to the formulation is demonstrated, creating a reproductible NLC. In (**C**,**E**) the different test concentrations with NaDES A can be observed, which can be also verified for NaDES B in (**D**). In (**F**), the reproducibility of the nanocarrier with the addition of NaDES is verified. (**G**) exposes the different conductivity potential between both the nanocarriers with and without NaDES.

**Figure A2:**
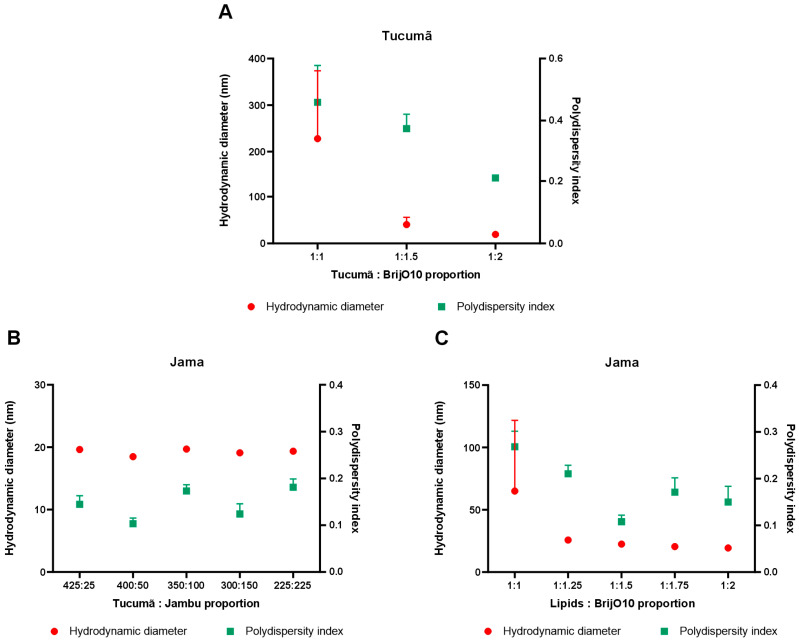
In (**A**), the test with different Brij^®^ O10 proportions in the SLN can be verified. In (**B**), the results obtained with different formulation proportions of butter and oil are shown. With the addition of oil to the formulation, the usage of Brij^®^ O10 could be reduced by 50%, as can be verified in (**C**).

Figure 1A shows the results obtained in the temperature test with the already established tucumã-based nanocarrier, proving that the temperature of 80 °C yielded the best DH and PDI results for the purpose of the work, which indicates a better homogeneity of the formula. Figure A2A shows the test with different proportions of surfactant, justifying the maintenance, initially, of a 1:2 ratio of butter and Brij^®^ O10, respectively.

Although the 400:50 and 300:150 ratios of butter to oil showed even better results that the chosen proportion (225:225 the of butter to oil), as shown in Figure A2B, it was assumed that the greater the amount of oil in the formulation, the more bioactives, drugs, and other products of choice could be stored using this technology [9]. The tests combining the lipids showed that their ZetaSizer parameters remained close to the solo tests made, as can be verified in Figure 1B. In Figure A2C, it is noted that the results of the different proportions tests concluded that the 1:1 ratio of lipid to Brij^®^ O10 yielded formulations with good PDI and DH values, even though other proportions had more promising results. This surfactant proportion was already proven viable in other works of the group [8]. This chosen proportion is also an advantage for further tests, since the less surfactant the sample has, the less toxic the technology becomes [10]. Thus, it can be noted that the combination of tucumã butter and jambu oil made it possible to reduce the amount of Brij^®^ O10 in the mixture by 50%, greatly reducing the possible cytotoxic nature of the technology due to the high initial surfactant content. At the end of this round of experiments, Jama was improved to its best formulation.

The two NaDESs have different proportions of water. Both have equal molar proportions of malic acid and betaine, which were 1:1. Comparing Figure 1C and Figure 1D, it can be verified that the first one demonstrated more promising results when evaluating DH and PDI parameters, which led this NaDES to be chosen to further tests.

The 45% NaDES A was chosen to compose the formulation of JamaNaDES since it presented the best parameters for a viable application in the future, as can be verified in Figure 1E. It is important to remember that, with the selection of this NaDES concentration in the formulation, the amount of water was reduced in the formulation. This reduction in water was a great outcome in this study, since the idea was to create a greener technology for industries based on the sustainability principles. Also, this reduction affects the high water usage in laboratories. Triplicate results can be seen at Figure 1F, which confirm the reproducibility of the technology. It is necessary to have in mind that, when diluted with water, an NaDES’ physicochemical properties can be tailored in a controllable way [11].

The zeta potential and conductivity differences between the nanocarriers occur due the presence or absence of NaDES in the formulations, which helps in the understanding of the solvent behavior and the intermolecular and intramolecular bonds that happen, as can be verified in Figure 1G. A relation between the shift in pH of nanocarriers when NaDES is added and the shift from negative to positive zeta potentials was already seen in the literature [12].

**Figure 2:**
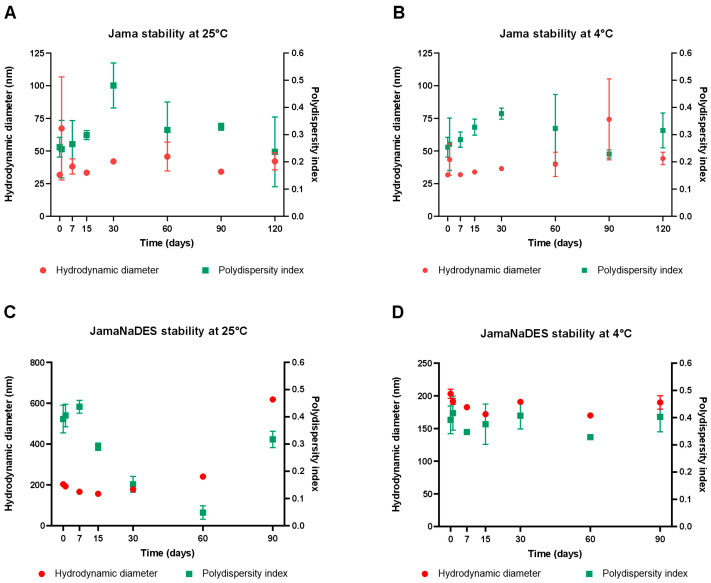
In (**A**,**B**), the results from the stability test for Jama at room temperature (25 °C) and refrigerator temperature (4 °C), respectively, are shown. (**C**,**D**) shows the results from the stability test for JamaNaDES also at room temperature (25 °C) and refrigerator temperature (4 °C), respectively.

Stability tests were performed for Jama and JamaNaDES at room temperature (25 °C) and refrigerator temperature (4 °C), both from 0 to 90 days of storage. In Figure 2A,B the DH and PDI results obtained of Jama at room temperature are shown, while Figure 2B shows the same parameters, but for Jama maintained at refrigerator temperature. Figure 2C shows the results for the DH and PDI of JamaNaDES at room temperature, while Figure 2D shows the same parameters for JamaNaDES maintained in the refrigerator. It can be observed that the JamaNaDES sample maintained at 4 °C shows a smaller variation in DH and PDI compared with the JamaNaDES sample stored at room temperature and the Jama nanocarriers stored in these two same environments. NLCs are already known for having an enhanced stability compared to SLNs [9], but the addition of NaDES to the nanocarrier formulation and its better results at 4 °C can also be due to NaDES’ cryoprotectant properties [4].

**Figure 3:**
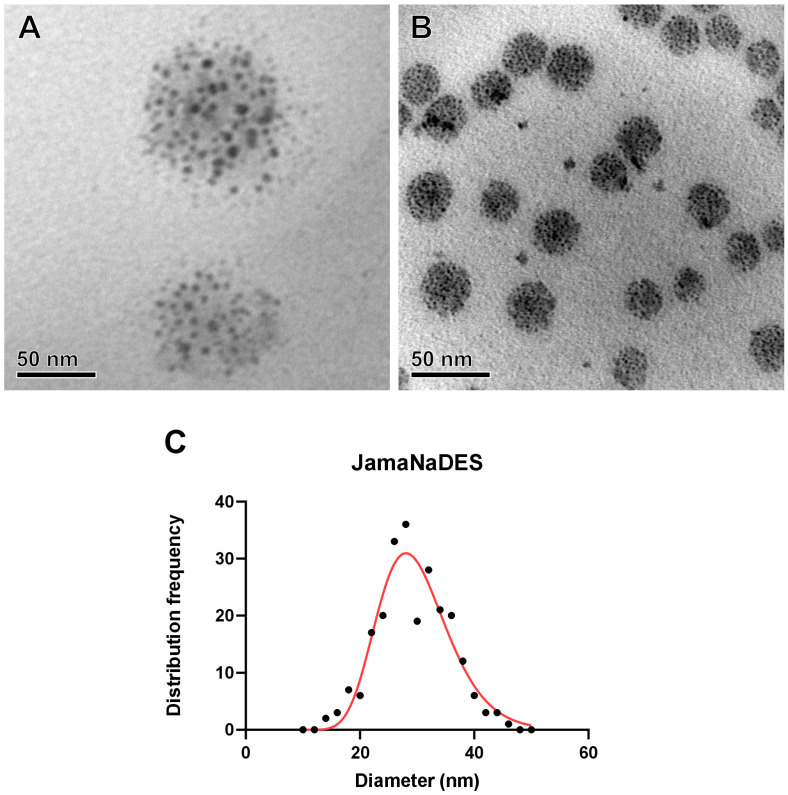
NLC ultrastructures and size distribution. Transmission electron micrographs of Jama are shown in (**A**) and of JamaNaDES in (**B**). In (**C**), the size distribution frequency of the particles in the images is shown. The nanostructures observed were measured, and the frequency distribution of observed diameters of JamaNaDES are presented in (**C**), with the red line representing the log-normal distribution. The TEM images of the nanocarrier were made in order to validate the formulation of the NLC, to study the morphology, and to help to elucidate of the behavior of the formation. The technology did not require osmium contrast, as the analysis was performed using conventional protocols since the tucumã butter has diverse electron-dense components that contrast the image [13].

A nanocarrier with a well-constituted spherical shape was observed in both formulations. Figure 3A shows Jama, and Figure 3B shows JamaNaDES. In Figure 3C, it is possible to see the size distribution frequency of the particles in the TEM photos.

### 2.2. Deciphering the Novel Formulation: In-Depth Look at the NaDES-Enriched NLC

IR spectroscopy was utilized to assess the alterations induced in nanocarrier samples due to either (i) the maintenance of water content (following a conventional process) or (ii) the reduction in water content with the addition of the solvent formulated.

- *NaDES and its constituents*

**Figure 4:**
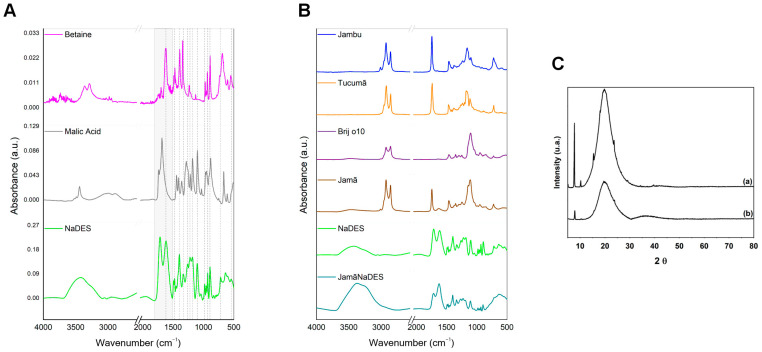
Figure (**A**,**B**) demonstrates the results of the IR spectrum. The NaDES and its constituents, betaine and malic acid (**A**), and the lipidic phase of the nanocarriers Jama and JamaNaDES are shown, along with NaDES spectrum profile (**B**). Diffractograms of the lipidic nanocarriers produced can be observed in (**C**): (a) Jama (absent of NaDES) and (b) JamaNaDES.

The IR spectra pertaining to NaDES and its constituents, betaine (C_5_H_11_NO_2_) and malic acid (C_4_H_6_O_5_), provide successful evidence of the solvent formulation, given the distinction between their spectral profiles and similarities (Figure 4A). The dashed lines along the spectral acquisition window (4000 to 500 cm^−1^) serve as characteristic indicators of the NaDES. Within this range, several peaks are shared among the samples analyzed (Table 1), situated around 1616.37 (νas(COO-)), 1452.24 (νas(CO_2_)), 1396.48 (ν(CN)), 1332.83 (δ(NCH)), 1220.96 (ϭ(CH_2_)), 1180.45 (ν(CO)), 1101.37 (ν(COH)), 983.98 (r(CH_2_) and/or ν(C-OH)), 932.44 (δ(CCN)), 892.25 (ν(CC)), 717.53 (δs(HCN)), and 601.80 (ρs(HOH) (x)) cm^−1^.

Variations induced by the influence of betaine and malic acid are notable in the NaDES spectral range between 3600 and 2600 cm^−1^, primarily associated with the presence of hydroxyl (ν(OH)) bands/peaks and methylene groups (CH_2_), along with evident shifts in certain signatures ranging between 1790 and 1500 cm^−1^ (highlighted in light grey, featuring strong and well-defined peaks). The constituents also affect the shapes and intensities of absorption bands/peaks and give rise to a double peak in NaDES (1712.81 and 1616.37 cm^−1^) attributed to the carboxylic acid group.

- *Formulated NLCs*

The incorporation of the NaDES into the NLC formulation, featuring a lipidic phase (Table 2) comprising jambu oil, tucumã butter, and Brij^®^ O10, is further supported by the spectra illustrated in Figure 4B. In these spectra, the NaDES and the nanocarrier formulated with its inclusion (JamaNaDES) exhibited striking similarities (Table 3), with multiple and well-defined peaks. The majority of them, within the range of 1500 to 840 cm^−1^, are found at comparable positions, presenting variations in relative intensity, shape, and/or broadening. This region is influenced both by the functional groups of the constituents of NaDES (betaine and malic acid) (Figure 4A) as well as by the groups of compounds present in the lipidic phase of the nanocarrier (Figure 4B) corresponding to hydrocarbons/amines (-CH_2_(N-CH_3_)), methylene (CH_2_), carboxylate (COO-), carboxylic (C=O), and alcoholic (C-OH) groups, in addition to carbonate vibrations (CO_2−_) and aliphatic and aromatic esters (C=O and C-O), among others (Table 1, Table 2 and Table 3). The differentiation between the two samples (NaDES and JamaNaDES) occurs in the regions from 3700 to 2690 cm^−1^ and from 850 to 500 cm^−1^. Markedly, in JamaNaDES, a discernible rise in relative intensity is noted (around 3369.69 cm^−1^), accompanied by a downward shift in frequency and a broadening of the band associated with hydroxyl groups when compared to the spectrum of NaDES isolated. Such behavior typifies robust hydrogen bonding and interactions with other molecules involved in the reaction, resulting in band overlap.

In contrast to JamaNaDES, the Jama sample (formulated with water only and in the absence of NaDES) exhibits a spectral profile with clear similarities to the lipidic precursors (oil, butter, and tensioactive). This resemblance (between the lipidic part and the NLC produced) was previously reported in the literature [14], and the peaks/band can be primarily attributed to the presence of methylene groups, indicated by a prominent double peak at 2922.20 and 2852.76 cm^−1^, and aliphatic esters, characterized by a strong narrow peak at 1743.67 cm^−1^, also present in the butter and oil spectra. Additionally, the peak around 1109.08 cm^−1^ corresponds to the characteristic band of Brij^®^ O10, a surfactant derived from vegetable-based fatty ethers of lauryl (NaC_12_H_25_S0_4_), cetyl (C_16_H_34_O), stearyl (C_18_H_38_O), and oleyl (C_18_H_36_O) alcohols. With this, it is suggested that the presence of pure water did not induce significant changes within the produced nanocarrier.

The X-ray diffractometry technique was applied to evaluate changes in the NLC crystallinity profile induced by the inclusion of NaDES in the formulation. The results presented in Figure 4C depict the diffractograms within the range of 5 to 80° at 2θ, comparing both the nanocarriers produced. In Jama (Figure 4C (a)), sharp peaks at 5.15°, 7.73°, 10.20°, 15.38°, and 23.88° are identified and attributed to the crystallinity of the constituents of tucumã butter, such as lauric and myristic acids [15], which comprise the majority of its composition [16]. Conversely, a noticeable presence of a broad peak around 20° is observed in JamaNaDES (Figure 4C (b)), potentially associated with a less ordered arrangement of the nanocarrier formulated with NaDES constituents [17]. This indicates that the solvent modified the homogeneity of the structure, resulting in a change in the crystalline profile towards an amorphous state. Such structural disorganization is consistent with the literature and may influence the incorporation of active ingredients into the nanosystem, thereby facilitating their diffusion and enhancing the encapsulation capacity of the nanosystem and its stability during storage [16].

### 2.3. In Vitro Cell Viability Tests on L132 Human Fibroblasts and Zebrafish Embryotoxicity Tests

**Figure 5:**
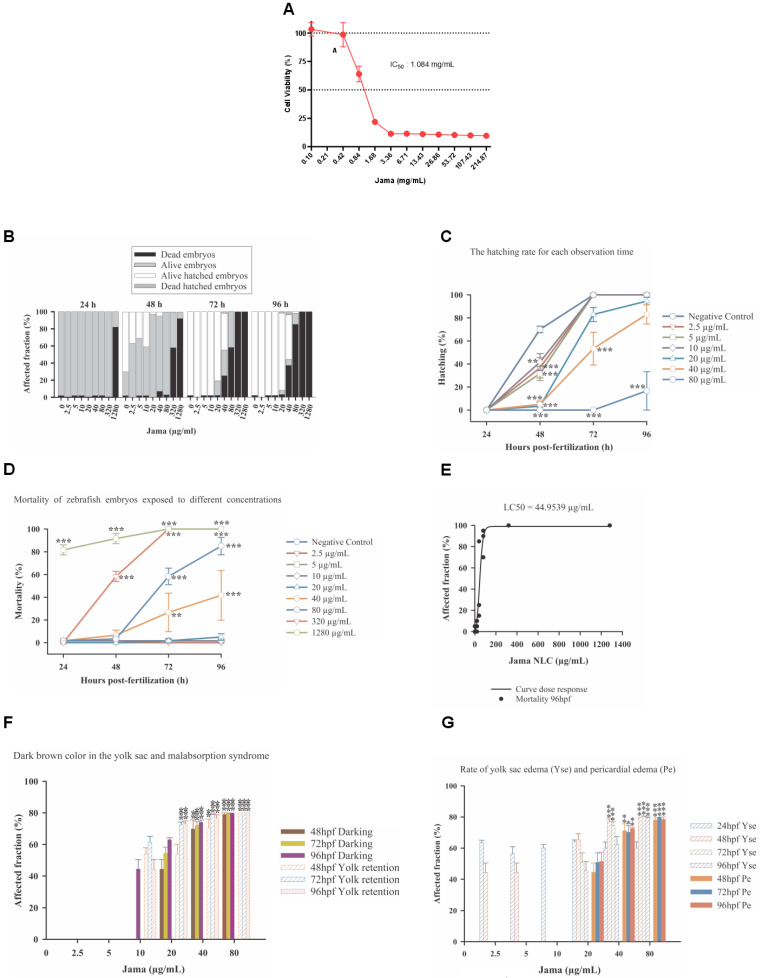
The cell viability as assessed by MTT assay after treatment with different concentrations of JamaNaDES for 24 h can be observed in (**A**). L132 cells were plated at a density of 1 × 10^4^ cells per well and treated with varying concentrations of JamaNaDES ranging from 214.87 mg/mL to 0.10 mg/mL. Following treatment, cells were incubated with MTT for 4 h, and absorbance was measured at 595 nm emission using a microplate reader. Cell viability values were calculated relative to the untreated control and expressed as a percentage. In (**B**), an overview graph of the Jama embryotoxicity test can be observed. In (**C**), the embryo hatching rates of zebrafish exposed to different concentrations of Jama for each observation time (hpf) are shown. Data are presented as mean ± standard error. Asterisks indicate significant differences between treatments and the control for a given time period (determined by Dunnett’s post-hoc comparison, *p* < 0.01 **; *p* < 0.001 ***). In (**D**), the mortality of zebrafish embryos exposed to different concentrations of Jama from 24 to 96 hpf is shown. Data are presented as mean ± standard error. Asterisks indicate significant differences between treatments and the control for a given time period (determined by Dunnett’s post-hoc comparison, *p* < 0.01 **; *p* < 0.001 ***). Dose-response curve (mortality) of organisms exposed to Jama for 96 h—LC50 44.9539 µg/mL—Model: sigmoidal—four parameters. R2 0.93 can be verified in (**E**). The graph of significant sublethal effects identified during the 96 h exposure to Jama can be found in (**F**). Data are presented as mean ± standard error. Asterisks indicate significant differenced between treatments and the control for a given time period (determined by Dunnett’s post-hoc comparison, *p* < 0.01 **; *p* < 0.001 ***). The rates of yolk sac edema (Yse) and pericardial edema (Pe) caused by Jama are shown in (**G**). Asterisks indicate significant differences between treatments and the control (determined by Dunnett’s post-hoc comparison, *p* < 0.05 *; *p* < 0.01 **; *p* < 0.001 ***). Error bars indicate standard error.

Initially, we conducted cell viability tests using L132 human fibroblast cells. Upon exposure to JamaNaDES, a significant decrease in cell viability was observed, establishing an inhibitory (cytotoxic) concentration of 50% (IC50) at 1.084 mg/mL (Figure 5A).

To assess the impact of Jama in an in vivo model, we performed zebrafish embryotoxicity tests. This assay provides comprehensive insights into the potential teratogenic or toxic effects of the compound during early developmental stages. Using the baseline concentration of 1.084 mg/mL, the IC50 observed in L132 fibroblasts, zebrafish embryos were exposed to Jama and monitored over a defined period. The results of this test are presented below, offering valuable information about the safety of the Jama formulation in a broader biological context.

During the 96 h test period, no significant lethal (mortality) or sublethal effects (changes in behavior, hatching, heart rate, and embryonic development) were observed for the negative control (cultivation system water—physical—chemical characteristics previously described). Additionally, the negative control exhibited normal development as described by Kimmel et al. in 1995 [18].

It was observed that the different concentrations of Jama (stock solution concentration = 22.5 mg/mL) were homogeneous at the time of dissolution and remained so until the end of the test. Figure 5B provides an overview of the embryotoxicity test. The tested concentrations were: 0 (negative control, with system water), 2.5 µg/mL, 5 µg/mL, 10 µg/mL, 20 µg/mL, 40 µg/mL, 80 µg/mL, 320 µg/mL, and 1280 µg/mL, and the total exposure time was 96 h.

A statistical analysis of hatching data over the 96 h test period was performed (Figure 5B). The statistical analysis examined the effects of two independent variables, “hours” and “concentration”, as well as their interaction. Initially, tests for normality and equality of variance were conducted to validate the suitability of the data for analysis of variance (ANOVA). Although the data did not pass the normality test, indicating a potential violation of the normality assumption, they satisfied the equality of variance test (*p* = 0.164). ANOVA revealed significant main effects for both “hours” and “concentration”, as well as a significant interaction between them, indicating that both factors and their interaction had a substantial impact on the dependent variable (embryo hatching). This was supported by significantly high F values and very low *p* values, suggesting a significant difference between groups and a very low probability of results being due to chance. Least squares means analyses and subsequent multiple comparisons provided a detailed understanding of differences between levels of each factor, highlighting specific combinations that showed statistically significant differences. Concentrations of 320 µg/mL and 1280 µg/mL were excluded from the tests, as there was 100% mortality on the third day of testing.

Therefore, it is evident that the increase in the concentration of Jama interfered with embryo hatching, in addition to the interference of time. It is possible to identify that at the three lowest concentrations (2.5 µg/mL, 5 µg/mL, 10 µg/mL), despite having a significant difference at 48 hpf, all surviving embryos were able to hatch by 72 hpf. For embryos in the control group and treatment groups with 20 µg/mL and 40 µg/mL, hatching began at 48 hpf (Figure 5B), and almost all surviving embryos hatched by 72 hpf and 96 hpf (Figure 5B,C). Compared to that of the control, the hatching rate of embryos in test solutions containing 80 µg/mL of the nanocarrier was significantly lower throughout the exposure time, with hatching only starting at 96 hpf (Figure 5B,C) for some surviving embryos, indicating more a significant inhibition of hatching in embryos exposed to higher doses of Jama. No hatching was observed at concentrations of 320 µg/mL and 1280 µg/mL (Figure 5B).

The analysis employed a two-way analysis of variance model to investigate the effects of the variables “hours” and “concentration” on the mortality rate. A significant interaction between these factors was found, suggesting that the effect of different hours post-fertilization on mortality depends on the concentration used (Figure 5D). This result highlights the importance of considering the interaction between factors in similar studies, as conclusions about the effect of one factor may be influenced by the level of the other factor. Additionally, multiple comparisons revealed significant differences between concentration groups at all hours post-fertilization, indicating that the concentration of the evaluated compounds plays a crucial role in the observed mortality rate.

The estimated value for x0 (44.9539) is crucial, as it represents the concentration of the nanocarrier at which the biological response reaches half of its maximum magnitude. In other words, it is the lethal concentration (LC50) that kills 50% of the population under study (Figure 5E). Furthermore, an additional statistical analysis indicated a good fit of the model to the data, with an R-squared coefficient of determination of 0.93. Normality and constant variance tests confirmed that our data met the necessary assumptions for the application of the regression model, increasing the reliability of our conclusions. Therefore, based on these findings, we can state that the average lethal concentration (LC50) of Jama is close to 44.9539 µg/mL, providing a solid basis for future investigations into its toxicity and biological effects.

Essentially, the analyses showed that both exposure time and concentration have a significant impact on both absorption and alterations in the yolk sac of zebrafish, manifesting either as darkening or as the appearance of malabsorption syndrome. In other words, when these two factors are combined, they interact significantly, further affecting the outcomes of the zebrafish (Figure 5F).

In addition to the sublethal effects reported above, there was a significant increase in edemas, both in the pericardium (Pe) and in the yolk sac (Yse) (Figure 5G), at the higher concentrations (40 µg/mL and 80 µg/mL).

**Figure 6:**
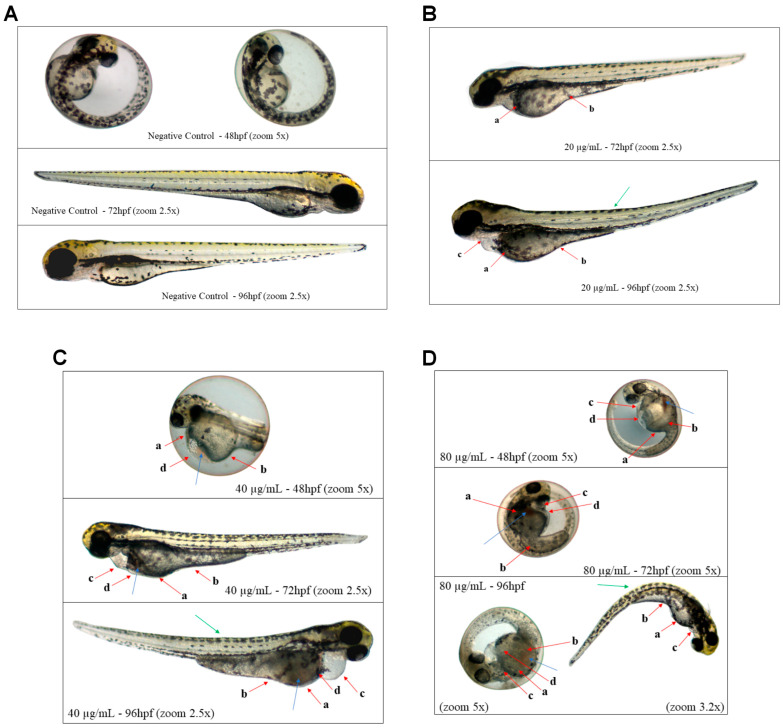
Photo documentation of organisms exposed for 96 h to concentrations of 0 mg/L (negative control), 20 µg/mL, 40 µg/mL, and 80 µg/mL of Jama can be observed in (**A**–**D**), respectively. Red arrows—a: darkening of the yolk sac; b: malabsorption syndrome; c: pericardial edema; d: yolk sac edema. Blue arrows—blood stasis. Green arrows—deformation in the notochord.

Thus, despite many sublethal effects not showing statistically significant differences, they were still identifiable in the individuals (Figure 6).

### 2.4. Investigating the Impact of Nanostructured Lipid Carriers on Matrix Metalloproteinase-9 Activity

Recent studies have unveiled a compelling interaction between NLCs and the activity of matrix metalloproteinase-9 (MMP-9) [19,20,21], a critical enzyme in the degradation of extracellular matrix components, directly acting on cell motility and tissue remodeling [22,23,24]. Notably, MMP-9 plays a pivotal role in various physiological and pathological processes, including wound healing, angiogenesis, and the metastasis of cancer cells [25,26,27]. The modulation of MMP-9 by NLCs, therefore, emerges as a significant area of interest, particularly in therapeutic contexts where such regulation is beneficial.

**Figure 7:**
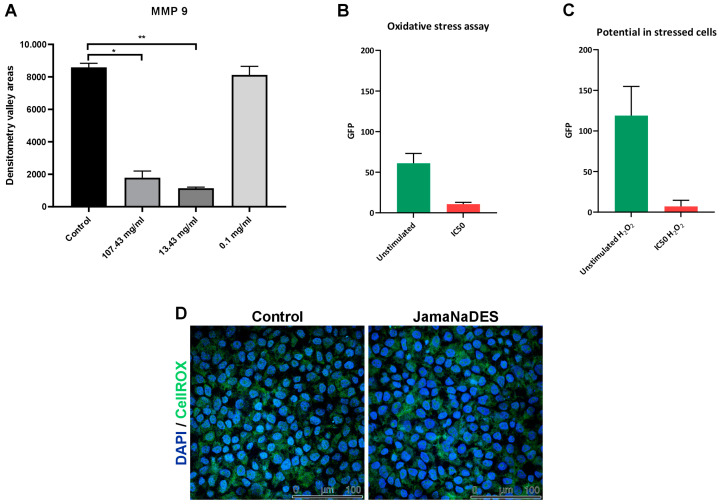
In (**A**), there is a graphic of the areas of the densitometry valleys for both the control group and the JamaNaDES treatment groups. The treatment group consists of the L132 cell supernatant treated with JamaNaDES at different concentrations. The asterisk indicates a significant difference between the groups (*p* < 0.05). In figure (**B**,**C**) the reactive oxygen species (ROS) production assessed using the CellROX^®^ Green Reagent assay after treatment with different concentrations of JamaNaDES for 24 h is shown. The data are presented as the raw values of GFP. In (**B**), there is a depiction of control cells and treated cells with ROS measurement, and in (**C**), there is the addition of H_2_O_2_ to observe action in a stressed environment. In (**D**), there are confocal fluorescence images of the experiment.

Our findings demonstrate a marked decrease in MMP-9 activity with the JamaNaDES treatment, indicating a potential inhibitory effect of the nanocarrier on this enzyme. In Figure 7A, it can be noted that the groups treated with higher concentrations of JamaNaDES differed significantly from the control group (*p* value = 0.0006 for 13.43 mg/mL and *p* value = 0.0025 for 107.43 mg/mL), while the groups treated with lower concentrations of the carrier did not (*p* value = 0.37 for 0.1 mg/mL), suggesting a dose-dependent interaction.

The ability of JamaNaDES to modulate MMP-9 activity, coupled with its demonstrated biocompatibility and low toxicity, as evidenced by cell viability and zebrafish embryotoxicity tests, positions it as a promising tool in the field of targeted therapy. JamaNaDES’s potential to serve as a delivery vehicle for therapeutic molecules aimed at regulating MMP-9 activity opens new avenues in the treatment of diseases characterized by aberrant MMP activity, including inflammatory and fibrotic disorders [28,29,30,31].

Further investigation into the mechanisms underlying the NLC-mediated regulation of MMP-9 is crucial. Understanding the interaction at a molecular level will elucidate the potential pathways through which JamaNaDES exerts its inhibitory effect. The clinical applicability and broader therapeutic implications of these findings will be a key focus of future studies, paving the way for the development of innovative, less invasive treatment strategies harnessing the unique properties of nanostructured lipid carriers.

### 2.5. Antioxidative Dynamics of NanoJama: Mitigating Oxidative Stress

For the present study, we chose to prioritize the use of native lipids from Brazilian flora, selecting tucumã butter and jambu oil both for their successful match in the formulation of the NLC and for their extremely important bioactive properties. Several relevant components have been found in tucumã, such as flavonoids, rutin, catechin, quercetin, carotenoids, riboflavin, and saturated fatty acids. It has also been proven that this fruit possesses antioxidant properties [13]. Jambu, on the other hand, contains a wide range of secondary metabolites attributed to anti-inflammatory and nociceptive properties involving opioid receptors in the process, along with many other interesting effects for industrial application [32,33].

The graphical data (Figure 7B,C) on the antioxidant activity of NLCs under oxidative stress and non-stressed conditions reveal compelling insights into their potential therapeutic applications. The fluorescence intensity in the “ROS with H_2_O_2_” graph indicates a marked increase in reactive oxygen species (ROS) in the presence of H_2_O_2_, a common inducer of oxidative stress, suggesting heightened cellular distress. Contrastingly, the significantly lower fluorescence bars in the “ROS without H_2_O_2_” graph suggest the NLCs’ potential to mitigate ROS, showcasing their inherent antioxidant properties. In Figure 7D, it is possible to see the images of confocal fluorescence of the control cells and the treated cells.

These observations may infer that the NLCs have an intrinsic ability to deliver antioxidant molecules efficiently, thereby quenching free radicals and ameliorating the oxidative stress in the cells. Such a reduction in ROS can be attributed to the intrinsic bioactive compounds within the formulation of the NLCs, which may function by either directly scavenging the free radicals or by modulating the cellular antioxidant defense mechanisms.

When it comes to the NLCs in this study, this feature is mostly associated with tucumã butter, which has in its composition flavonoids (the most important one being quercetin) and carotenoids, which are the most popular and well-known antioxidant compounds [34,35,36]. Other studies have already identified this effect by evaluating the cytoprotective effect of tucumã in lymphocyte cultures exposed to H_2_O_2_ through spectrophotometric, fluorometric, and immune assays. As was already described by other authors, the results confirmed the presence of β-carotene and quercetin, well-known antioxidants. The results showed an increase of the viability of cells exposed to H_2_O_2_ [36,37,38].

These findings present a case for NLCs as promising vehicles for enhancing the bioavailability and efficacy of antioxidant agents, potentially offering a targeted approach to treat diseases where oxidative stress is a contributing factor. However, the exact molecular mechanisms underlying the antioxidant effects observed require further investigation. It would be essential to elucidate whether these effects are due to the sustained release of antioxidants, the stabilization of sensitive compounds within the NLC matrix, or the induction of cellular pathways that bolster endogenous antioxidant defenses.

For future studies, it would be beneficial to investigate the NLCs’ performance against a range of oxidative stressors and to quantify their impact on different cell types. Such studies would not only validate the current findings but could also expand the scope of NLCs’ applicability in clinical settings, particularly for conditions exacerbated by oxidative stress. Additionally, exploring the relationship between the NLC composition, the loading capacity of antioxidant molecules, and their release kinetics could provide a more comprehensive understanding of the NLCs’ functionality as antioxidant delivery systems.

In conclusion, the presented data underscore the therapeutic potential of NLCs as antioxidants, warranting further exploration into their role as a novel modality in combating oxidative stress-related pathologies.

### 2.6. Tuning Nanoparticle Size via Crystallization and Its Industrial Implications

**Figure 8:**
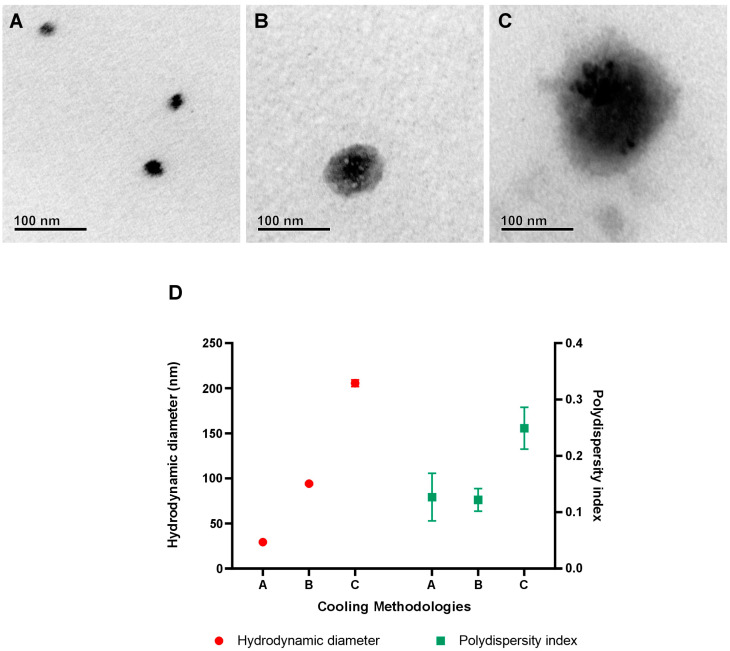
Morphological and physicochemical changes in Jama after different cooling methods. TEM micrographs of the three different cooling methods are observed in (**A**–**C**). In (**D**), the hydrodynamic diameter and polydispersity index are presented. In figure (**A**–**C**), the TEM microscopy of the nanoparticles obtained in protocols A, B, and C, respectively, are shown. Figure (**D**) displays the results obtained through the dynamic light scattering (DLS) technique, indicating the hydrodynamic diameters and polydispersity indices achieved using different cooling methodologies (A, B, and C).

Mastery over nanoparticle crystallization plays a critical role in the industrial scalability of nanotechnologies [39,40]. In this study, the cooling methods proved decisive in modulating the size of Jama with significant precision. Rapid cooling method A yielded nanoparticles with diameters of just 30 nm, while the slower, controlled method C produced particles with diameters up to 200 nm. Intermediate procedure B was capable of producing particles with sizes up to 95 nm. These results are shown in Figure 8D and underscore the potential for adapting NLCs for a variety of industrial applications, aligned with specific active delivery needs. TEM microscopy was also performed with the A, B, and C protocols and can be observed in Figure 8A–C, respectively.

The precision tuning of nanoparticle size can transform the efficacy and stability profile of products, directly influencing the specificity and efficiency of the delivery of molecules of interest [41]. In the industrial context, the ability to produce NLCs with controlled dimensions enables the exploration of new market niches and the enhancement of existing products, ranging from pharmaceutical formulations to advanced cosmetics [42]. The versatility of NaDES-formulated NLCs supports the industry’s commitment to sustainable practices and the development of green solutions, providing a pathway for less invasive and more natural manufacturing processes.

The role of the NaDES in modulating the homogeneity and transitioning from crystalline to more amorphous states was particularly noteworthy, indicating not just an improvement in the NLCs’ loading capacity but also potential for the optimization of active release. This, along with the ability to significantly reduce water usage in the formulation process and the toxicity of the carrier, aligns with emerging trends in industrial nanotechnology, where the demand for eco-efficient and biologically responsible production methods is growing.

The incorporation of green chemistry principles into NLCs highlights the potential for their application on a large scale, a significant advantage for industries interested in innovations that meet not only market demands but also sustainability imperatives. The data from this study emphasize the feasibility of customizing NLCs for a wide range of applications, establishing a new paradigm for industrial nanotechnology that is environmentally sustainable, adaptable, and effective.

## 3. Conclusions

While research and testing involving NaDESs and their integration with NLCs are still in nascent stages, the ongoing pursuit of less deleterious technologies to both the environment and human health must be steadfastly pursued. The capability of this new technology to deliver larger drug or bioactive volumes directly to target tissues, employing a less adverse solvent for organisms, represents a significant stride towards greener chemistry and industry. The incorporation of therapeutic properties inherent to the mixture’s components extends the adaptability of the technology across a spectrum of applications.

The newly associated NaDES-NLC emerges as a robust contender for a diverse array of applications, demonstrating the feasibility of merging greener, more natural processes with formulations in the pharmaceutical and cosmetics industries. The garnered results showcase that, comparatively, this technology can be as or even more effective than conventionally employed methods and synthetic components. Furthermore, the reduction in water usage in the formulation process and in carrier cytotoxicity, owing to the amalgamation of these technologies, is noteworthy.

## 4. Materials and Methods

### 4.1. Selection of Lipids

The study commenced with a screening test encompassing the following butters: bacuri (*Platonia insignis*), cacau (*Theobroma cacao*), cupuaçu (*Theobroma grandiflorum*), muru-muru (*Astrocaryum murumuru*), tucumã (*Astrocaryum aculeatum*), and ucuuba (*Virola surinamensis*). A screening test was also performed with the following oils: babaçu (*Attalea speciosa*) and bacaba (*Oenocarpus bacaba*). Both oils and extracts were tested for buriti (*Mauritia flexuosa*), copaíba (*Copaifera langsdorffii*), jambu (*Acmella oleracea*), mulateiro (*Calycophyllum spruceanum*), palmiste (*Elaeis guineensis*), and urucum (*Bixa orellana*). All of the cited lipids are native Brazilian flora and are seen as by-products by the industries. This research received authorization from the National System for the Management of Genetic Heritage and Associated Traditional Knowledge (SisGen) under the approval number A1563A6. These materials were chosen to be tested because their physicochemical characteristics and bioactive properties were verified as suitable for the formulation. Preliminary tests indicated that a 1:2 ratio of butter/oil to surfactant yielded standardized results. Tucumã butter and jambu oil demonstrated compatible physicochemical characteristics, suggesting their potential synergistic efficacy in a combined nanocarrier formulation.

### 4.2. Pre-Formulation Studies

The primary objective was to develop a nanocarrier platform using only the selected butter, starting from a well-established and stable SLN. Then, a temperature scan was conducted with tucumã butter and surfactant (Brij^®^ O10) at 1:2 ratios. Optimal results were observed at 80 °C, establishing this temperature as the standard for further formulation processes. At this cited temperature, the “cloud point” was observed. Cloud point extraction relies on the observation that aqueous solutions containing certain non-ionic surfactants exhibit clouding behavior when the temperature is adjusted accordingly. The cloud point, or critical temperature, is specific to the type and concentration of the surfactant. Beyond this temperature, phase inversion occurs due to the disruption of hydrogen bonds between the surfactant and water. After phase inversion, the mixture is cooled to temperatures well below the cloud point, facilitating the reestablishment of hydrogen bonds between the surfactant and water. This phenomenon induces interfacial turbulence, which is responsible for the formation of nanostructures [43].

### 4.3. Incorporation of Jambu Oil

Subsequent experiments involved testing different surfactant ratios while maintaining the original butter-to-surfactant proportion. With the butter-based nanocarrier optimized, varying ratios of butter to jambu oil were explored. A 1:1 ratio demonstrated the most promising outcomes. This formulation underwent triplicate testing to ensure reproducibility and consistency, followed by zeta potential measurement. This nanocarrier, comprising tucumã butter and jambu oil, is henceforth referred to as “Jama”.

### 4.4. Reduction in Surfactant Quantities

The subsequent phase focused on reducing surfactant quantities in the nanocarrier composition, aiming to minimize cytotoxicity. Various proportions were tested, and the nanocarrier was prepared in triplicate under the new protocol with the adjusted Brij^®^ O10 (oleyl alcohol polyoxyethylene ether is a bio-based, high-HLB, non-ionic surfactant from naturally occurring straight-chain oleyl alcohol) concentration. These confirmation tests affirmed the reproducibility and consistency of results.

### 4.5. Selection of NaDES

In the NaDES selection, a formulation that could be used in pharmaceutical products was prioritized. In Brazil, both malic acid and betaine have their use authorized by ANVISA (Brazilian Health Regulatory Agency). The authorization of malic acid use is cited in the Normative Instruction number 211 of 2023 [44]. Betaine authorization can be consulted in the Medication Consultation available in the ANVISA website [45].

Furthermore, malic acid and betaine already presented many useful properties. Both have anti-freezing properties and resistance to high osmotic pressure [3]. Betaine is successfully used as an extractant medium for antioxidant molecules [4,46]. Malic acid, on the other hand, has been used as an alternative extractant for the isolation of natural products from medicinal plants. Industrially, malic acid has been used as an anti-aging skin care agent [4,47,48].

In order to, for future perspectives, add an antioxidant active, it was decided that an NaDES composed of malic acid and betaine was the best choice.

### 4.6. NLC and NaDES Combination

Having optimized the NLC, two NaDES formulations were tested, NaDES A and NaDES B, both comprising malic acid, betaine, and water (in 1:1:4 and 1:1:7 molar ratios, respectively), based in the studies of Aryati et al. [49] and Mustafa et al. [50]. Initial tests were conducted in three experimentally pre-established proportions. NaDES A, with a molar ratio of 1:1:4 (malic acid, betaine, and water, respectively), exhibited superior performance. In this formulation, water was partially replaced by the NaDES. Higher NaDES concentrations in the formulation showed promising outcomes, leading to the selection of a nanocarrier with 45% NaDES for further study. This formulation underwent triplicate testing to confirm reproducibility and was designated as “JamaNaDES”.

### 4.7. Stability Tests

Stability assessments were conducted for both Jama and JamaNaDES under various storage conditions, including room temperature (25 °C) and refrigeration (4 °C). A Malvern Zetasizer Lab (Chicago, IL, USA), model ZSU3100, was employed to measure the polydispersity index (PDI), hydrodynamic diameter (DH), and zeta potential (PZ) of the nanocarrier.

### 4.8. Cultivation and Maintenance of L132 Cells

L132 cells were maintained in DMEM supplemented with 10% fetal bovine serum (FBS) and 1% antibiotics (penicillin–streptomycin). The cells were maintained in an incubator at 37 °C with 5% CO_2_.

### 4.9. MTT Cell Viability Assay

An MTT (3-(4,5-dimethylthiazol-2-yl)-2,5-diphenyltetrazolium) assay was performed to assess cell viability. Initially, L132 cells were plated in 96-well culture plates at a density of 1 × 10^4^ cells per well. After cells adhered, they were treated with different concentrations of JamaNaDES: 214.87 mg/mL, 107.43 mg/mL, 53.72 mg/mL, 26.86 mg/mL, 13.43 mg/mL, 6.71 mg/mL, 3.36 mg/mL, 1.68 mg/mL, 0.84 mg/mL, 0.42 mg/mL, 0.21 mg/mL, or 0.10 mg/mL. The treatment was maintained for 24 h in incubation at 37 °C with 5% CO_2_.

Following the treatment period, cells were carefully washed with PBS (1×) to remove any treatment residue. Then, fresh medium containing 5 mg/mL MTT was added, and cells were further incubated for 4 h under the same temperature and CO_2_ conditions. After MTT incubation, the culture medium containing MTT was removed, and DMSO was added to solubilize the formazan formed. Absorbance was measured using a microplate reader (Varioskan LUX Multimode Microplate Reader, ThermoFisher^®^, Waltham, MA, USA) at 595 nm emission. Cell viability values were calculated relative to the untreated control, expressed as a percentage of viability.

### 4.10. Reactive Oxygen Species (ROS) Production Assay

To evaluate reactive oxygen species (ROS) production, we employed the CellROX^®^ Green Reagent (ThermoFisher^®^, Waltham, MA, USA) assay at a concentration of 5 µM. After treating L132 cells with different concentrations of JamaNaDES, cells were divided into four groups: unstimulated, stimulated, stressed unstimulated, and stressed stimulated.

To induce oxidative stress, hydrogen peroxide (H_2_O_2_) was added at a concentration of 26 mM along with the treatment. The dose used was the IC50 obtained from the MTT cell viability assay.

Cells were incubated with the respective treatments for 24 h at 37 °C with 5% CO_2_. After the incubation period, cells were washed with PBS (1×) and incubated with the probe for 30 min at 37 °C.

Following probe incubation, cells were washed again with PBS (1×) to remove excess non-internalized probe. Intracellular fluorescence was then measured using a microplate reader (Varioskan LUX Multimode Microplate Reader, ThermoFisher^®^, Waltham, MA, USA) with excitation at 485 nm and emission at 520 nm at specified time intervals.

### 4.11. Confocal Microscopy Analysis

After treating L132 cells (1 × 10^3^ cells per well) with a concentration of 420 µM of JamaNaDES for 4 h, cells were carefully washed with PBS (1×) and fixed in 3.5% formaldehyde for 15 min at room temperature. Subsequently, cells were stained with DAPI (4′,6-diamidino-2-phenylindole) at a concentration of 500 nM for nuclear labeling and with CellROX^®^ Green Reagent (ThermoFisher^®^, Waltham, MA, USA) at a concentration of 5 µM for the detection of intracellular reactive oxygen species (ROS).

Images were acquired using a confocal microscope from Leica, model CS SP5, in different z-stacks to capture the three-dimensional structures of the cells. Wide-field images were obtained for the overall analysis of nuclear localization and CellROX^®^ Green Reagent (ThermoFisher^®^, Waltham, MA, USA) fluorescence throughout the cell population. Quantitative analyses were performed on CellROX^®^ Green Reagent fluorescence intensity.

### 4.12. Morphology of NLCs

The determination of NLC morphology was performed via transmission electron microscopy (TEM) using a JEOL 1011 instrument (Tokyo, Japan), which was operated at an accelerating voltage of 100 kV. The suspensions were diluted 1:10 (*v*:*v*) with Milli-Q^®^ water and deposited directly into the carbon-coated grids used for the observation of samples using osmium tetroxide. The microscope was operated in bright-field mode with a magnification above 10,000× for morphological analysis.

### 4.13. Infrared Spectroscopy

Infrared spectra were acquired using a Vertex 70 spectrometer (Bruker Corporation, Billerica, MA, USA) in attenuated total reflectance (ATR) mode. Samples in both liquid and solid states were analyzed, with liquid samples (2 µL) undiluted and powder samples placed directly on a diamond crystal. Spectra collection ranged from 4000 to 500 cm^−1^ at 4 cm^−1^ resolution, averaging 64 scans in absorbance mode. Data acquisition was managed with OPUS 7.2 software (Bruker Corporation, Karlsruhe, Germany), and subsequent processing utilized Origin Pro 2015 software (OriginLab Corporation, Northampton, MA, USA).

### 4.14. Zebrafish Embryo Culture

The zebrafish embryos utilized in the embryotoxicity test were supplied by the Zebrafish Facility (Techniplast, Buguggiate, Italy) of the Laboratory of Toxicologic Genetic of University of Brasília. Adult zebrafish were maintained in an automatized recirculating water system, stocked with water filtered by activated coal. The physical and chemical characteristics of the water were maintained at pH 7.2–7.6, hardness 6.70 dH, temperature of 26 ± 1 °C, and conductivity of 728 μS. The facility room was maintained with a photoperiod of 12:12 h light/dark. The fish were fed two or three times a day with commercial feed (SERAVipan^®^; Tetramin^®^, Blacksburg, VA, USA) and live feed (Artemia salina nauplii).

### 4.15. Toxicity Test with Zebrafish Embryos (FET)

The embryo tests were based on the OECD protocol n.236 for toxicity assessment: Fish Embryo Toxicity—FET test [51]. After collecting the embryos from the aquariums, they were washed and immediately distributed onto microplates with “test solutions” to ensure the beginning of exposure in the early stages. Exposure was carried out in 96-well microplates with 200 µL of each concentration. The tests were conducted in a climatic chamber with conditions identical to those of the culture room. The test solutions were prepared using zebrafish culture water (physical and chemical characteristics previously described). All tests were performed in triplicate with a total of 60 organisms per concentration and for 96 h.

### 4.16. Zymography

Zymographic analysis was performed to evaluate the activity of specific proteases in a polyacrylamide gel copolymerized with enzyme substrates. This experiment focused on matrix metalloproteinases (MMPs) degrading gelatin in the gel, creating colorless bands. The procedure adhered to protocols by Hawkes et al. [52], utilizing L132 cell supernatants treated with the JamaNaDES formulation at varying dilutions [25,52]. A molecular marker (ThermoScientific PageRuler Prestained Protein Ladder) was used for MMP identification by molecular weight. Gels were scanned on an ImageQuant LAS 4000 (GE Healthcare, Chicago, IL, USA) and analyzed using ImageJ 1.54 software (National Institute of Health, Bethesda, MD, USA).

### 4.17. Statistical Analysis

Data were statistically analyzed using Sigma Plot 14.0. For normally distributed datasets, one-way ANOVA was used to detect intergroup differences. In cases of non-normal distributions, the Kolmogorov–Smirnov test for normality and Levene’s test for variance homogeneity were applied, followed by the Kruskal–Wallis test. Significant differences between tested concentrations and controls were determined using Dunnett’s or Dunn’s test for parametric or non-parametric data, respectively (*p* < 0.05).

## Figures and Tables

**Table 1:** IR peaks and their assignments for betaine, malic acid (MA), and NaDES samples.

Solvent			
Frequencies (cm^−1^)			Assignments
Betaine	MA	NaDES	
-	3439.13	-	ν(O-H)
-	-	3417.91	ν(OH)
3359.22	-	-	ν(OH)
3286.01	-	-	ν(OH)
-	3019.50	-	νs(CH(CH_3_))/ν(OH)
2980.02	-	-	ν(CH(CH_2_))
2946.29	-	-	δs(HOH)
-	2883.62	-	νs(CH_2_)
-	1737.89	-	ν(C=O)
-	-	1712.81	ν(C=O)
1695.10	1688.99	-	ν(C=O)
1621.72	-	1616.37	νas(COO-)
-	-	1490.30	νas(-CH_2_(N-CH_3_))
1467.96	-	1474.08	νs(COO-)
-	1440.84	1452.24	νas(CO_2_)
-	1409.43	-	δ(COH)
1391.96	-	1396.48	ν(CN)
-	1357.90	-	νs(CO_2−_)
1339.54	-	1332.83	δ(NCH)
-	1284.50	-	ν(CO)
-	-	1259.53	-
1232.96	-	1220.96	ϭ(CH_2_)
-	1177.92	1180.45	ν(CO)
1126.38	-	-	δ(CCO)
-	-	1101.37	ν(COH)
-	1099.44	-	ν(COH)
-	1031.93	1043.39	ν(COH)
-	-	983.98	ϭ(CH_2_)/
972.62	966.51	-	ν(COH)
-	951.66	954.28	ν(COH)/ δ(CCN)
934.18	-	932.44	δ(CCN)
892.25	881.48	892.25	ν(CC)
-	747.23	-	ρs(HOH)/ ν(COH)
-	-	717.53	δs(HCN)
687.82	-	-	-
-	665.45	-	ν(COH)
-	-	636.51	-
603.08	607.58	601.80	ρs(HOH) (x)

ν (stretching), νas (asymmetric stretching), νs (symmetric stretching), δ (bending), ρ (rocking) and ϭ (scissoring).

**Table 2:** IR peaks and its assignments for Tucumã butter, Jambu oil and Brij^®^ O10 samples.

Lipid			
Frequencies (cm^−1^)			Assignments
Tucumã	Jambu	Brij^®^ O10	
-	3008.27	-	ν(CH)
2918.14	2922.20	2927.32	νas(CH(CH_2_))
2849.98	2852.76	2852.76	νs(CH(CH_2_)
1734.75	1744.96	-	νs(C=O) aliphatic
1463.07	1462.06	1463.49	ϭ(CH_2_)
1380.02	1377.19	-	δs(CH) out-of-plane/ϭ(CH_3_)
-	-	1349.80	ϭ(CH_3_)
-	-	1293.53	-
1237.25	1236.39	1249.93	ν(CO)/δ(CH_2_)
1176.47	-	-	ν(C=CCO)/ν(CO) aliphatic/δ(CH_2_)
1158.46	1159.23	-	
1108.93	-	1102.31	ν(CO)
-	1098.38	1099.93	ν(CO)
-	1033.86	-	ν(CO)
-	-	949.10	δ(CH=CH)
-	-	847.80	νs(CCO)
721.98	721.38	723.98	ρ(CH_2_)

ν (stretching), νas (asymmetric stretching), νs (symmetric stretching), δ (bending), ρ (rocking), and ϭ (scissoring).

**Table 3:** IR peaks and their assignments for Jama, JamaNaDES and NaDES samples.

NLC			
Frequencies (cm^−1^)			Assignments
Jama	JamaNaDES	NaDES	
3477.66	-	-	ν(O-H)
-	-	3417.91	ν(O-H)
-	3369.69	-	ν(O-H)
3007.07	-	-	ν(O-H)
2922.20	-	-	(CH(CH_3_))/ν(OH)
2852.76	-	-	ν(CH(CH_2_))
1743.67	-	-	νs(C=O) aliphatic
-	1716.67	1712.81	ν(C=O)
1627.94	1624.08	1616.37	νas(COO-)
-	1492.75	1490.30	νas(-CH_2_(N-CH_3_))
1463.99	1474.74	1474.08	νs(COO-)
-	1453.36	1452.24	νas(CO_2_)
-	1398.41	1396.48	ν(CN)
1347.63	-		ϭ(CH_3_)
-	1334.76	1332.83	δ(NCH)
1298.39	-	-	-
-	1267.25	1259.53	-
1244.75	-	-	ν(CO)/ δ(CH_2_)
-	1224.87	1220.96	ϭ(CH_2_)
-	1189.97	1180.45	ν(CO)
1141.87	-	-	νs(CO_2−_)
1109.08	1105.22	1101.37	ν(COH)
-	1042.52	1043.39	ν(COH)
989.49	979.49	983.98	ϭ(CH_2_)/ν(COH)
948,99	956.98	954.28	ν(COH)/δ(CCN)
-	929.96	932.44	δ(CCN)
-	895.07	892.25	ν(CC)
843.80	-	-	νs(CCO)
721.38	713.67	717.53	δs(HCN)
-	630.78	636.51	-
-	607.59	601.80	ρs(HOH) (x)

IR frequencies in cm^−1^ of the nanocarrier and NaDEs samples.

## Data Availability

Data is contained within the article.

## Appendix A

The figures here are represented in the text as A1-x and are cited in the “Results and Discussion” section. Figure A1 represent the DLS tests with all the butters and oils selected for primary research. In Figure A2, supplementary tests for the formulation of the SLN, the NLC, and Brij^®^ O10 adjustments are verified.
